# New and Pre-existing Eating Disorders Among Adolescents and Young Adults During the COVID-19 Pandemic: A Population-Based Cohort Study

**DOI:** 10.23889/ijpds.v9i5.2625

**Published:** 2024-09-18

**Authors:** Amreen Babujee, Hong Lu, Therese Stukel, Natasha Saunders, Astrid Guttmann, Paul Kurdyak, Simone Vigod, Longdi Fu, Alene Toulany

**Affiliations:** 1 ICES; 2 The Hospital for Sick Children; 3 The Centre for Addiction and Mental Health; 4 Women's College Hospital

## Objective

Our understanding of the contribution of new presentations versus pre-existing eating disorders during the COVID-19 pandemic is limited. This study aims to evaluate rates of emergency department (ED) visits and hospitalizations for eating disorders among adolescents and young adults (YA) new to care and those with pre-existing eating disorders during the pandemic.

## Approach

We conducted a population-based cross-sectional study using linked health administrative data for Ontario residents aged 10-26 during the pre-pandemic (Jan. 1, 2017-Feb. 29, 2020) and pandemic periods (Mar. 1, 2020-Jun. 30, 2022). We used Poisson generalized estimating equations models to predict expected overall and monthly rates of eating disorder-related ED visits and hospitalizations among those with a new and pre-existing eating disorder.

## Results

Compared with expected rates, ED visits increased during the pandemic among only adolescents with new eating disorders (adolescent RR 2.12, 95% CI [1.84,2.45]). Additionally, both adolescents and YA with pre-existing eating disorders experienced an increase in ED visits (RR 2.78, 95% CI [2.28, 3.38] and RR 1.52, 95% CI [1.25, 1.85], respectively). Similarly, hospitalizations for new presentations increased solely for adolescents (RR 1.48, 95% CI [1.34,1.64]), while hospitalizations for pre-existing eating disorders increased for both adolescents (RR 1.82, 95% CI [1.43,2.32]) and YA (RR 1.12, 95% CI [1.01,1.23]).

## Conclusions

There was an increase in acute care visits for eating disorders during the pandemic, especially among adolescents and YA with pre-existing conditions. This differentiation is important in advancing our understanding of the pandemic's effects on adolescents and YA and the healthcare system receiving them.

